# ATAQS: A computational software tool for high throughput transition optimization and validation for selected reaction monitoring mass spectrometry

**DOI:** 10.1186/1471-2105-12-78

**Published:** 2011-03-18

**Authors:** Mi-Youn K Brusniak, Sung-Tat Kwok, Mark Christiansen, David Campbell, Lukas Reiter, Paola Picotti, Ulrike Kusebauch, Hector Ramos, Eric W Deutsch, Jingchun Chen, Robert L Moritz, Ruedi Aebersold

**Affiliations:** 1 Institute for Systems Biology, Seattle, WA, USA; 2 Institute of Molecular Systems Biology, ETH Zurich, Zurich, Switzerland; 3 Information Warehouse, the Ohio State University Medical Center, OH, USA; 4 Competence Center for Systems Physiology and Metabolic Disease, ETH Zurich, Zurich, Switzerland; 5 Faculty of Science, University of Zurich, Zurich, Switzerland

## Abstract

**Background:**

Since its inception, proteomics has essentially operated in a discovery mode with the goal of identifying and quantifying the maximal number of proteins in a sample. Increasingly, proteomic measurements are also supporting hypothesis-driven studies, in which a predetermined set of proteins is consistently detected and quantified in multiple samples. Selected reaction monitoring (SRM) is a targeted mass spectrometric technique that supports the detection and quantification of specific proteins in complex samples at high sensitivity and reproducibility. Here, we describe ATAQS, an integrated software platform that supports all stages of targeted, SRM-based proteomics experiments including target selection, transition optimization and post acquisition data analysis. This software will significantly facilitate the use of targeted proteomic techniques and contribute to the generation of highly sensitive, reproducible and complete datasets that are particularly critical for the discovery and validation of targets in hypothesis-driven studies in systems biology.

**Result:**

We introduce a new open source software pipeline, ATAQS (Automated and Targeted Analysis with Quantitative SRM), which consists of a number of modules that collectively support the SRM assay development workflow for targeted proteomic experiments (project management and generation of protein, peptide and transitions and the validation of peptide detection by SRM). ATAQS provides a flexible pipeline for end-users by allowing the workflow to start or end at any point of the pipeline, and for computational biologists, by enabling the easy extension of java algorithm classes for their own algorithm plug-in or connection via an external web site.

This integrated system supports all steps in a SRM-based experiment and provides a user-friendly GUI that can be run by any operating system that allows the installation of the Mozilla Firefox web browser.

**Conclusions:**

Targeted proteomics via SRM is a powerful new technique that enables the reproducible and accurate identification and quantification of sets of proteins of interest. ATAQS is the first open-source software that supports all steps of the targeted proteomics workflow. ATAQS also provides software API (Application Program Interface) documentation that enables the addition of new algorithms to each of the workflow steps. The software, installation guide and sample dataset can be found in http://tools.proteomecenter.org/ATAQS/ATAQS.html

## Background

Proteomics aims at the comprehensive identification and quantification of the proteins present in a biological sample. Typical samples include lysates of cells, tissue extracts, body fluids such as serum or plasma, or fractions of complete proteomes such as organelles or subcellular fractions. These samples usually contain thousands to tens of thousands of different proteins and their complete analysis has been technically challenging, in spite of significant recent progress. Most proteomic measurements have been carried out by mass spectrometry. Several strategies have been developed that all involve the generation of a protein sample, the digestion of the proteins, typically with trypsin, and the separation, ionization and mass spectrometric analysis of the complex peptide samples. There are several strategies for mass spectrometry-based experiments [1,2]. In the most commonly used strategy, referred to as data dependent analysis (DDA), shotgun proteomics or discovery proteomics, the instrument samples specific precursor ions (molecular ions of intact peptides) from all the precursor ions detected in a survey scan using a simple heuristics. Even though the sampling rate on modern mass spectrometers has increased considerably over the last few years, for complex proteome samples, the number of precursor ions detected in a survey scan typically exceeds the number of selection and fragmentation cycles in the instrument. Consequently, with repeat analyses of identical or very similar samples, different subsets of peptides are identified, resulting in irreproducible data sets.

Recently, a complementary proteomic workflow has emerged that is based on the targeted analysis of a set of predetermined proteins and peptides. This workflow is based on a mass spectrometric method referred to as selected reaction monitoring (SRM). It involves the selection of proteotypic peptides[3,4] from the predetermined protein set and the targeted selection of precursor ions based on their mass to charge ratio, the fragmentation of the precursor ions in the collision cell of a QQQ mass spectrometer and the selective detection of peptide-specific fragment ions. The detected fragment ions derived from a specific precursor ion are referred to as transitions [5]. The precursor ion mass and the corresponding optimized set of transitions, along with additional information such as the preferred charge state of a peptide ion and the chromatographic elution time of the peptide, constitute a specific and highly sensitive assay for the detection of a particular peptide in a sample. SRM-based mass spectrometry produces consistent, reproducible and highly sensitive data sets that are particularly important for comparison of protein profiles across multiple samples, as is the case with biomarker discovery and validation studies and in systems biology where a biological system is analyzed in differentially perturbed states [6,7].

**Figure 1:**
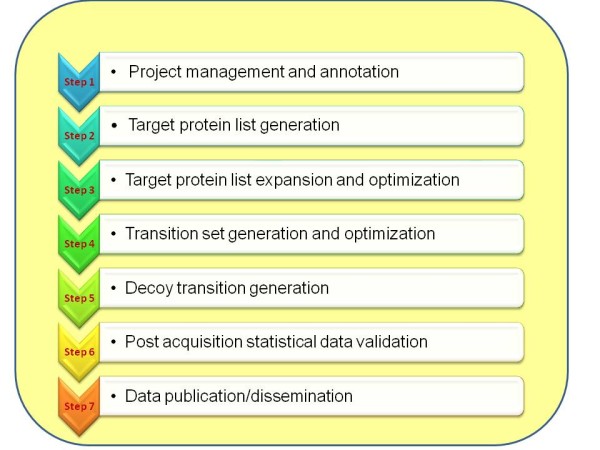
**Summary of ATAQS workflow**. ATAQS is composed of seven steps. The data flow is flexible in that the user can select which steps they want to use in cases in which the whole pipeline is not used. For example, if the user has already generated and optimized the list of transitions and decoy transitions, then the user can skip steps 4 and 5. In each step, there are major options for user to select or define. In step 1, the experiment needs to be annotated by selecting exact mass spectrum instrument and organism. Also, the user can select other researchers who can share the project. In step 2, the user needs to provide or select a list of proteins. In step 3, the user can explore the selected proteins' properties and interactions by using PIPE2. Then, the user can extend the protein list or trim it down to a smaller number of proteins. In step 4, the user needs to define what type of peptides and transitions to be selected for a given protein by specifying penalties of amino acid compositions and fragment ion types. In step 5, the user will select to generate a decoy or heavy/light pairs based on user-selected decoy generating algorithms and labeling methods. In step 6, mzXML or mzML format of measured data set is selected and the user groups the experiments by transition list. Then, user can also select transition property measuring algorithm in this step. Based on the results of step 6, the user can choose a FDR cutoff to determine validated peptides in a given sample. In step 7, as an option, the user can create a TraML format of verified transitions to share with the community.

Over the last decade, a rich environment of open source and proprietary software tools has emerged to support all aspects of shotgun proteomics. In contrast, software tools to support the targeted, SRM-based workflow are still sparse. Previously published TIQAM [8] software suites (TIQAM-digestor, TIQAM-peptidealtas, TIQAM-viewer) were also designed to support SRM workflow by generating transitions from in-silico digestion, by connecting to peptideatlas for transition selection and by manual examination of SRM triggered MS2 spectra. However, TIQAM connection to PeptideAtlas is limited when user wishes to prioritize peptides based on weighted amino acide composition. TIQAM-viewer is used for manual validation with assistant of MS2 spectra that are acquired by SRM triggered MS2 and there is no systematic method to classify "validated" vs. "not validate". Moreover, TIQAM is a single user desktop application rather than deployable and sharing the data in collaborative working environment. Similarly, Bertsch *et al. *recently published an algorithm to predict proteotypic peptides, their fragmentation and retention time using sequence information alone[9]. The recently published Skyline [10], a window client application, provides a way to build SRM methods based on the BiblioSpec, NIST and GPM formats of spectral libraries. Skyline also provides a quantification value by calculating the area under the curve using CRAWDAD software[11]. However, it uses a single score (hydrophobicity value from SSRCalc) to provide confidence in identification. Similar to TIQAM, it's a desktop application with manual inspection for validation. As a complementary tool to Skyline, AuDIT[12], which is a webserver application running at the GenePattern website, provides further statistical validation in quantification using user-provided quantification values of light and heavy transitions. However, there is no open or commercial software that supports the entire SRM workflow for multiple users and that can interface with web browsers on a personal computer as well as connect to institution-wide computing resources for high throughput data analysis. Here we introduce a new open source software pipeline, ATAQS (Automated and Targeted Analysis with Quantitative SRM), which provides modules with algorithms that collectively support all of the steps of the SRM assay development and deployment workflow for targeted proteomic experiments (Figure 1). ATAQS software is designed to support multiple users at an institution. ATAQS can be easily extended and customized by the user with the addition of user-implemented algorithms at any of the workflow steps. It also provides API for connecting to existing web service tools for easy data export and import to user's institutional web-based ATAQS application.

In this manuscript, we describe the following workflow steps of ATAQS that collectively support the routine application of SRM-based targeted proteomics studies:

1. ATAQS system overview

2. Workflow overview

3. Target Protein set selection

4. Peptide transition selection

5. Addition of isotopic pair and decoy transitions

6. Identification of confirmed transitions

7. Publication of verified transitions

In the section 'ATAQS application', we will also illustrate the ATAQS workflow with SRM-based analysis of synthesized yeast peptides and signaling kinase proteins in a human cancer cell line.

The ATAQS software is essential for the implementation and wide dissemination of a targeted proteomic workflow and therefore, this software is expected to have wide application in all fields of life science research that require the high-throughput generation of hypothesis-based, reproducible and highly sensitive proteomic datasets.

## Implementation

### ATAQS System Overview

**Figure 2:**
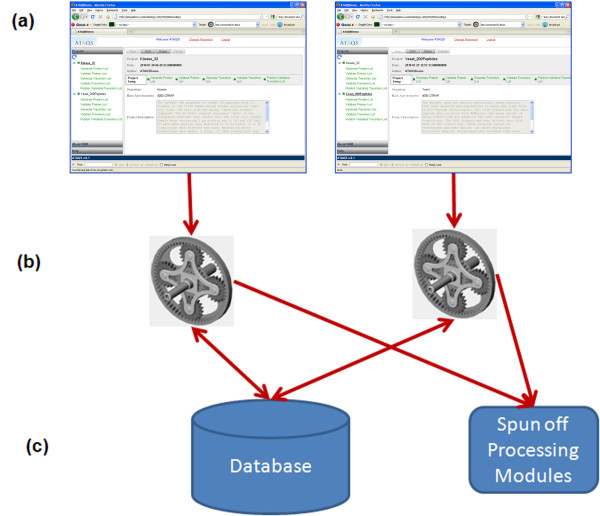
**ATAQS System Overview**. The ATAQS system is composed of three tiers: (a) shows a presentation tier. User interface ATAQS using Mozilla FireFox browser in any computer that can connect to ATAQS server. (b) illustrates a business logic tier. Servlet Coordinates the applications, by launching processing modules in processing system and interface with Database to store/retrieve data (c) illustrates the data storage and processing tier. Data are stored and retrieved and CPU heavy processing modules are running in a distributed CPU node system for high throughput.

ATAQS is written in Java and has a three-tier architecture to serve multiple users throughout an institution. ATAQS uses Google Web Toolkit (GWT) technology to create client and server codes and Hibernate to handle database transactions (Figure 2). The presentation tier (Figure 2a) provides a graphic user interface for the popular browser Mozilla Firefox. ATAQS utilizes Firegoose [13] in the business logic tier (Figure 2b) to connect to web-based service sites, representing the data storage and processing tier, (Figure 2c) such as PIPE2 (Protein Information and Property Exploration) [14] and PeptideAtlas [15] in order to utilize both in-house developed algorithm support, public databases and processing servers. Since ATAQS was designed to serve multiple users, it has a light version of LIMS to organize project lists and status by a given user and also to enable sharing of projects among user-specified members.

When a user is logged in as an administrator, the admin page will be displayed (Additional File 1: Admin section). The admin page allows the administrator to add users, a list of available organisms and types of mass spectrometers. When the admin chooses to add a mass spectrometer, ATAQS opens a current list of mass spectrometers from the EBI website (http://www.ebi.ac.uk/ontology-lookup) to acquire the standard description for the instrument.

The list of projects and their status for a logged-in user are indicated in the Projects panel on the left side. Shared projects from other members are read-only projects for the logged-in user. When logged in, the user can use the 'share' button, and ATAQS will list available users with whom the user can share the currently selected or created project in the project setup step (Additional File 1: Project setup section).

### Workflow Overview

ATAQS has six panels. First, the user will start with the 'Project Setup' panel to describe the project, and define the organism under study and the type of mass spectrometer used. Then, the user populates the protein list from a user-defined list or an institution-wide database in the 'Generate Protein List' panel. Optionally, the user can refine the candidate protein list using the 'Validate Protein List' panel. From the final list of proteins, proteotypic peptides, which are unique sequences for a protein and also typically observed by MS, and the corresponding transitions for each targeted peptide are obtained from the 'Generate Transition List' panel. Given the transition list and type of mass spectrometer specified for the measurement, ATAQS offers a selection of algorithms to generate a score and error estimate to measure the accuracy of the transition identification in 'Validate Transition List'. Finally, the user can choose to create a proteomics standard initiative format of the verified transition, referred to as TraML (Transition Markup Language)[16], through which generated transitions and data can be easily published or transferred to a public MRM transition website.

### Target protein selection and validation

SRM is typically used to measure candidate protein biomarkers or proteins for which are targeted in biological studies based on prior knowledge of the system, e.g. proteins for which the corresponding mRNA transcripts have been identified as being differentially expressed in transcriptome analyses of samples in different states. Therefore, the first task of ATAQS is to acquire a protein candidate list and to optimize the list by filtering or adding proteins to the initial candidate list. This optimization is accomplished by integrating other pertinent information for the proteins on the initial target list such as protein-protein interactions, cellular functions or associations with signaling networks. The user can upload their own protein list in the 'Generate Protein List' step or use administrator-installed databases which are available for all users of the institution, or merge user-based lists with available databases.

Currently, ATAQS provides three curated disease-specific protein candidate lists: (1) Prostate tumor, containing 1055 proteins, (2) Type II diabetes, containing 954 proteins and (3) Breast cancer-related human kinase signaling, containing 32 proteins. The prostate tumor and type II diabetes protein databases are bioinformatically curated using Medgene and the NIC GEO (Gene Expression Omnibus) Microarray differential expression analysis and human protein-protein interaction map from HPRD (Human Protein Reference Database). Numerous studies have confirmed the dysregulation of protein kinases in several disease, in particular cancer[17]. Based on the results of previous experiments and the current literature, we selected 32 protein kinases to target via SRM in the context of a breast cancer profiling study.

**Figure 3:**
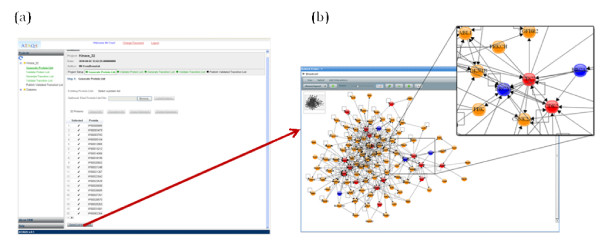
**PIPE2 connection**. ATAQS provides an automatic connection to the PIPE2 web server. Figure (a) shows the location of the simple button to activate the PIPE2 connection and (b) illustrates the network viewer in PIPE2 for protein-protein interactions associated with the protein list from ATAQS using HPRD.

The protein list from 'Generate Protein List' (or the uploaded user-defined protein list) can be exported to the PIPE2 website. PIPE2 includes various pipelets such as ID Mapper and network visualization that allow users to filter or add proteins from the protein list broadcast by the ATAQS 'Generate Protein List' step. Figure 3 illustrates the PIPE2 network visualization. The curated protein list can be broadcast back to ATAQS and is listed in the 'Validate Protein List' panel. This optimized or refined target protein list is the basis for the following targeted proteomic analyses.

### Peptide transition selection

Given a refined/optimized protein list, the next step is to obtain proteotypic peptides for each protein. The proteotypic peptide set can be defined as a unique peptide set that can be used to unambiguously identify the target set of proteins in biological samples. Desirable proteotypic peptides are sequences unique to a given protein that are also observed by mass spectrometry with MS2 spectra that can be assigned to the respective peptide sequence with high confidence. Thus, ATAQS relies on PeptideAtlas, which contains millions of identified spectra to obtain proteotypic peptides and the corresponding transition selections. The PABST (Peptide Atlas Best SRM Transition) webpage generates the optimal peptide and transition lists.

PABST performs two sequential selections based on user-defined criteria. First, peptides are selected primarily based on the likelihood of observation and the presence or absence of various sequence features. To address the former, we first query the PeptideAtlas database and find all peptides mapping to a subject protein that have been observed more than once. We also generate a list of all possible tryptic peptides from this same protein (in silico digestion using TIQAM), and apply two separate peptide detectability algorithms, Peptide Sieve and Peptide Detectability Predictor. If the empirical proteotypic score is available, it will be used, otherwise, the theoretical proteotypic score is assigned. The peptide is then evaluated based on user-defined criteria, such as number of amino acids (peptide length), amino acid composition and uniqueness of sequence (does not map to more than one protein or one region in the genome). A user-weighted factor score is then calculated and the final score is calculated by combining the empirical proteotypic score and the user-weighted factor score. The list of peptides is sorted by this score in descending order and the user-defined number of peptides is selected.

**Figure 4:**
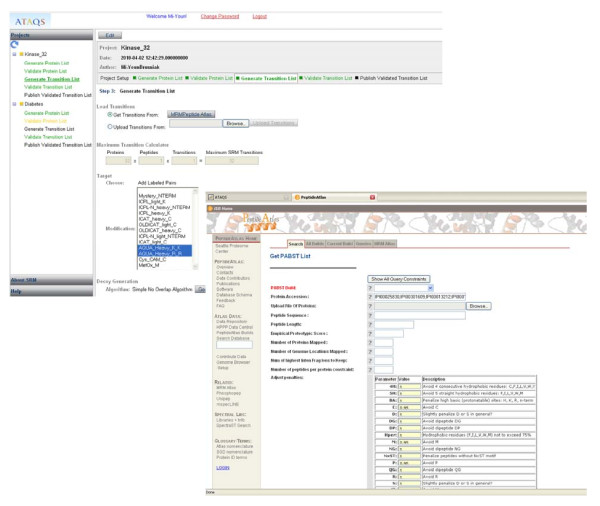
**Transition list**. ATAQS sends the protein list to the Peptide Atlas Best SRM Transition website and generates the best peptide set based on an empirical score that uses a user-defined penalty weight. For example, the user can increase the penalty value to 2 to avoid a methionine in the peptide sequence.

The next step is to generate a transition list. The best fragment ions for a given peptide are obtained in this step. First, SRM-verified transitions are deposited in MRM PeptideAtlas[18], which contains data from both the literature and from user submissions. If the requested number of transitions for a given peptide has not yet been met, the process looks at data from consensus spectral libraries, as these have information about which fragment ions are known to be generated in CID, as well as their relative intensities. If a triple quadrupole (QQQ) library is available, it is scanned first for the peptide of interest. Next, a QTOF library is consulted, if available, followed by an ion-trap library, which is generally the most complete. For a given spectrum that matches the subject peptide, peaks are taken in order of descending relative intensity. If there are no observed spectra for the subject peptide or the minimum number of requested transitions has not been met, additional theoretical fragments are determined based on TIQAM in silico digestion, which populates the list first with singly charged y-ions with m/z greater than precursor m/z, then with Y ions less than precursor m/z, and finally with B ions, if necessary. As more verified transition are deposited in the MRM PeptideAtlas, more transitions will be observed and retrieved in this step for a given mass spectrum. Figure 4 shows the PABST link from the ATAQS protein list broadcast.

Alternatively, the user can download transitions broadcasted from PABST to their desktop and add or modify peptides (e.g., PTMs or other modification peptides) and its related transitions. Then, the user can upload the extended transition list to the transition selection step by selecting the "Upload transitions from" option instead of the "Get transitions From: MRMPeptideAtlas" option.

It's also worth noting that MRM PeptideAtlas will have an option available in the near future for user's to choose instruments for selecting transitions along with the algorithm describe above (QQQ type first, then QTOF, etc.).

The final transition list is sent back from the PABST website to ATAQS by Firegoose. PABST is a public website with a fixed URL, while ATAQS is an institute-owned web application, rather than a web-based service outside of the institute's firewall (unless established as such by the administrator). Therefore, the user must add the ATAQS URL to receive the results (Additional File 1: Setting up connection to other website section). As an additional step, users can download the verified transitions generated in this step and can further optimize the transitions using their instrument setting. The confirmed transitions then can be uploaded back to this step to repopulate the transition for the following step.

### Adding isotopic pair and decoy transitions

It is common to use internal standards consisting of stable isotope-labeled reference peptides in the SRM workflow. These reference compounds facilitate accurate quantification and are useful for increasing the confidence in detecting the targeted peptides in complex samples. ATAQS provides a way to append various isotopic pair transitions to the given transition list in the 'Generate Transition List' panel. The current version of ATAQS uses the mProphet [19] validation algorithm which requires both a decoy transition measurement and the true target transition measurement to distinguish between true and false identifications of the targeted peptides. Therefore, after candidate peptides and transitions are obtained from PABST or via user uploads from other sources, there is an option to expand the transition list by adding user-specified isotope pair and decoy transitions. The current distribution of ATAQS provides two algorithms for generating decoy transitions. One is called 'Simple Algorithm' by which decoy transitions are generated using reversed peptide sequence and transitions based on the TIQAM-Digestor algorithm. Then we filter the decoy transitions by the same ion type and select the closest Q3 m/z value of the target transition. The recommended algorithm is called 'Simple - No Overlap Algorithm' by which decoy transitions are first generated in the same way as with 'Simple Algorithms', but then we add 10 Thomson to the decoy Q1 values. This approach generates the same number of decoy transitions and target transitions. The combined transitions and decoy transitions can be downloaded to the desktop of the user for consecutive mass spectrometry runs.

### Identification of confirmed transitions

**Figure 5:**
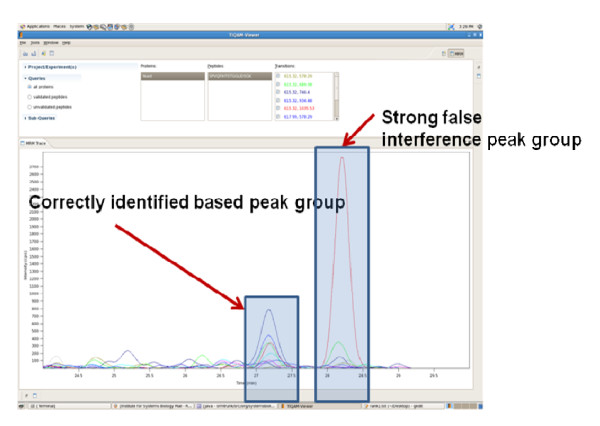
**ATAQS validator**. The ATAQS validator best peak group selection algorithm is capable of selecting the correct peak group when a strong false interference peak group is present.

The 'Validate Transition List' panel in ATAQS requires mzXML or mzML files from an SRM mass spectrometry run, along with a transition list that is generated by ATAQS. When transitions are measured across various background samples (e.g., different patients or different growth conditions for cells, etc.), the transitions need to be grouped according to the run ID. An example of a run ID assignment is explained in the case study section 'ATAQS application'. The trace for each detected transition is extracted and smoothed using DFT (Discrete Fourier Transformation). The smoothed transition traces are then used by the 'transition group algorithm' to select the best peak group called in ATAQS. For a given peptide we detect all transitions that belong to the peptide (Q1) and detect all the peaks of each transition that are above background noise. There are several transition peaks in a given transition trace. Each peak of a transition trace is then grouped with the nearest peak of the entire transition set for a targeted peptide. Then, for each peak group, the values of the following properties are calculated: (1) sum intensities, (2) retention time deviation, and (3) number of transitions in a peak group. Based on the ranked score of the three property values, the best transition peak group is selected. Figure 5 illustrates an example of a transition set of a given peptide after applying smoothing algorithms and also indicates our correct peak group selection over strongly interfering transition peak groups. The selected best peak group of a specified peptide is then used to calculate the following classifiers for further discriminant analysis: (1) sum total intensities in log2 scale, (2) dot product of heavy and light pair, (3) standard deviation of retention time, (4) deviation from expected retention time, (5) number of detected heavy transitions, (6) number of detected light transitions and (7) number of matched heavy and light pair transitions used in the dot product calculation. The seven peak group property values of the best peak group per peptide are used as 'features' for the semi-supervised learning approach implemented in mProphet. The sum of the intensity values is used as the main feature and the other six values are used as secondary features. The entire set of transitions is divided into a training set and a test data set using 10-fold cross validation. Then, the linear discriminant analysis (LDA) finds the linear combination of features that best separates the target transitions and decoy transitions using the training dataset. A null distribution of false peak group identifications is estimated from the decoy data points. The weight of this distribution is estimated based on the left part of the target distribution in the region where almost only false identifications are expected. Based on this null distribution, a false discovery rate in dependence of the chosen cutoff is estimated. ATAQS displays target and decoy transition discriminant score distributions, FDR and sensitivity curve and ROC.

### Publishing verified transitions

For hypothesis-driven proteomics, SRM technology can be a robust and simple assay method to verify the detection and accurate quantification of proteins in various biological samples if there are known verified and optimized transitions available for the set of candidate proteins. However, optimization and validation of a transition set that corresponds with the proteins of interest can be labor-intensive and time-consuming. Therefore, we developed a model of ATAQS to automate the optimization and validation of transitions, thereby making this a high-throughput process. We expect that generating verified transitions for any protein can be a joint effort within the LC-MS-based proteomics research community. We worked with the HUPO PSI (proteomics standard initiative) to define a file format that can be widely accepted for better exchange of verified information. We proposed the TraML (Transition Markup Language) schema to PSI and it is currently under review. The TraML schema captures transition information from both small molecules metabolomics and from proteomics communities. The most recent information on TraML development can be found at http://psidev.info/index.php?q=node/405.

ATAQS also provides an easy way to create and publish user-verified transition lists in the 'Publisher Verified Transition List' step. ATAQS collects some basic required information such as author contact, etc. and automatically creates a TraML file for users to review (Additional File 1: Publishing validated transitions section). The TraML file contains user-verified transitions for the selected project by selecting a cutoff discriminant score. Then, the user can upload the reviewed TraML file and use the 'publish' button in order to upload the TraML file to the MRM PeptideAtlas.

The above section describes the seven ATAQS steps that guide biologists or analytical chemists through the SRM experimental workflow. We attempted to make the ATAQS software architecture user-friendly, not only to users of the GUI (graphical user interface), but also to computational biologists who would like to add their own algorithms. New algorithms or modules can be written in java or other programming languages that can be executed by servlet executor codes. The detailed information regarding the servlet classes that are needed for an additional algorithm is provided in the ATAQS installation guide and software API documentation. When the system admin adds new extended algorithms to the server, the ATAQS GUI option will populate the new algorithm automatically. For the power users, it's worth noting that the CPU-intensive modules can be run by command line Linux shell scripts generated by ATAQS servlet codes. ATAQS servlet executors distribute the CPU intensive modules to many different nodes, and these modules are referred to as the 'spun off processing modules' in Figure 2.

## Results and Discussion

ATAQS also provides a flexible workflow so users can start and end at any of the seven steps. More specifically, the user can just use the transition validation step or just use the protein validation step. The following case studies highlight this flexibility of ATAQS, its mode of operation and its performance. The first example of the use of synthetic yeast peptides for SRM measurements illustrates the steps used to generate and validate transitions in the ATAQS pipeline, and the second example concerns the measurement of protein kinases in a human breast cancer cell line.

### Applying a subset of steps from ATAQS for detecting synthetic yeast peptides in a complex peptide matrix

The 'Validate Transition List' step is one of the critical steps for high throughput generation of verified transitions. In an effort to develop algorithms and software for describing the properties of each transition and for performing the statistical analysis, we prepared a test case study in which a set of chemically synthesized peptides of known sequence was diluted into a complex peptide background. Each target peptide was synthesized in isotopically heavy and light form to generate a distinctive transition signature for the discrimination of true from false signals.

One hundred proteotypic yeast peptides were selected and synthesized in heavy and light form by JPT Peptide Technologies, Inc [20]. Using the 'Transition Generator Panel' in ATAQS, the user can perform the fourth (Peptide transition selection) and fifth (adding isotopic pair and decoy transitions) steps described in the method section. Thus, for each peptide, five transitions were selected from the MRM PeptideAtlas, totaling 500 transitions that are based on MS2 data generated on an ABSCIEX 4000 instrument. Then, using the transition generator, the heavy pair transitions for the 500 transitions were added and a matching number of decoy transitions were added to the transition list, as described in the 'Adding isotopic pair and decoy transition' section. The resulting 2000 transitions were then downloaded to the user's desktop from ATAQS and divided into four sets of 500 transitions each. These transitions were measured on an ABI-Qtrap 4000 in SRM mode in samples containing the targeted peptides in three concentrations (1x, 4x, 64x) in three different complex peptide backgrounds consisting of N-glycopeptides isolated from human plasma and tryptic digests of C. elegans and Leptospira interrogans extracts, respectively. In total, 36 (3 biological backgrounds, 3 step dilution series times 4 sets of 500 transition lists) LC-MS SRM experiments were performed.

**Figure 6:**
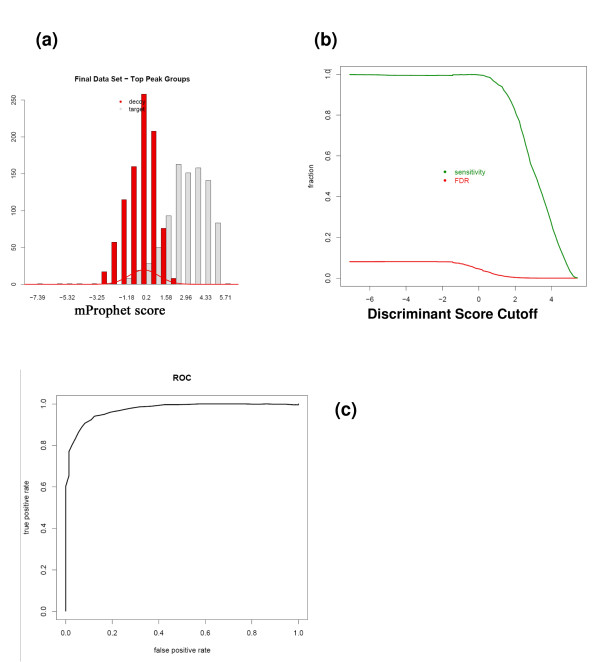
**Statistical Summary**. ATAQS provides a graphical summary report. This figure illustrates the results from the yeast 18000 transition SRM measurements. (a) shows the separation of decoy and target transition distributions by a semi-learning algorithm and (b) shows the FDR and sensitivity curve with respect to the discriminant score cutoff. (c) shows the ROC curve for the data.

The acquired data from all 36 samples were converted to mzXML and uploaded to a server along with the 2000 transition list file generated by ATAQS to the 'Validate Transition List' panel in ATAQS. The user then assigned 9 sample IDs based on the four LC-MS runs using the four subsets of the 2000 transitions list. More specifically, one dilution with one biological background was measured with the four subsets of the original 2000 transitions, resulting in four mzXML files. The four mzXML files were then assigned one run ID. As described in the 'identification of confirmed transitions' method section, the best transition peak group was determined for each measured peptide. Therefore, there were nine (3 different background times 3 dilutions) measurements for a target peptide in this example (nine Sample IDs). The best transition peak group list of 18000 transition measurements (9 samples times 2000 transitions) was generated and analyzed for statistical validation analysis. The summarized results are displayed in ATAQS in chart format with FDR and sensitivity curve and ROC (Figure 6). Figure 6(a) illustrates the good separation of target and decoy transitions. From the chart in Figure 6(b), the user can use the discriminant score cutoff of -1 to obtain a 5% FDR level. The 18000 transition list along with discriminant score can be downloaded to the user's desktop from the ATAQS 'Validate Transition List' step.

Figure 5 shows the correctly identified transition groups from this yeast experiment. This example illustrates how the user can confidently identify transitions from ambiguously identified transitions for a given FDR. The discriminant score of the100 peptides in each of the nine samples are provided at http://tools.proteomecenter.org/ATAQS/ATAQS.html.

### Applying ATAQS for detecting kinase proteins in a human breast cancer cell line

The second example set was prepared to target 50 peptides from 32 protein kinases in tryptic digests of extracts of the T-47D human ductal breast epithelial tumor cell line. The cell line was grown and lysed and the lysate was digested with trypsin. Fifty heavy-labeled reference peptides corresponding to PTP's of the targeted kinases were spiked into the total cell lysate. Sample sets containing 2 μg digested protein mass and 8, 40 and 200 fmol of each of the isotopically labeled reference peptides were measured in triplicate. The kinase protein list is also included in the ATAQS database. The project description was annotated in the ATAQS 'Project setup' and the protein list was populated in the 'Generate Protein List' step. We also investigated the candidate protein list in the context of protein networks using PIPE2 (Figure 3). PIPE2 provides utilities to explore functionalities, cellular location and protein-protein interaction for the candidate kinase proteins and notes additional protein candidates which can be pursued in future studies, e.g. by targeting mass spectrometry. The PIPE2 analysis indicated that our selected candidate proteins are associated with known cancer genes (e.g., BRCA). Eight to ten transitions were selected and equal amounts of decoy transitions were added. A total of 1896 transitions were divided into four transition sets. A total of 36 LC-MS SRM experiments (3 dilutions times triplicate runs times 4 sets of transitions) were performed. Both datasets were measured using the ABSCIEX 4000 QTRAP mass spectrometer. Human protein kinases were targeted by SRM from total human cell lysate and the data were analyzed using the entire ATAQS workflow.

**Figure 7:**
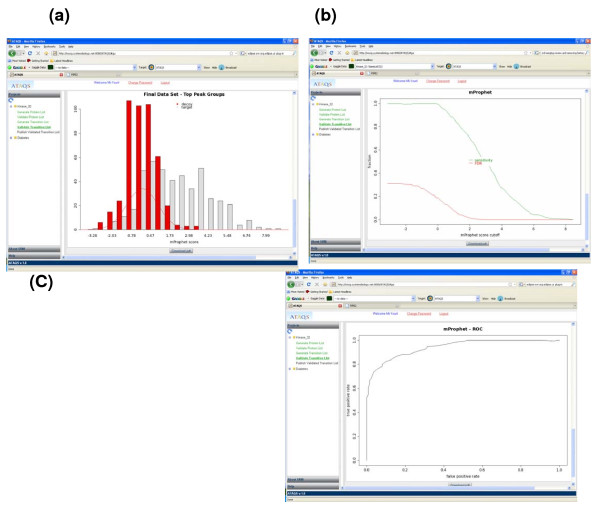
**Statistical summary of kinase dataset**. ATAQS provides a graphical summary report. This figure illustrates the results from the kinase transition SRM measurements. (a) shows the separation of decoy and target transition distributions by a semi-learning algorithm and (b) shows the FDR and sensitivity curve with respect to the discriminant score cutoff. (c) shows the ROC curve for the data.

**Figure 8:**
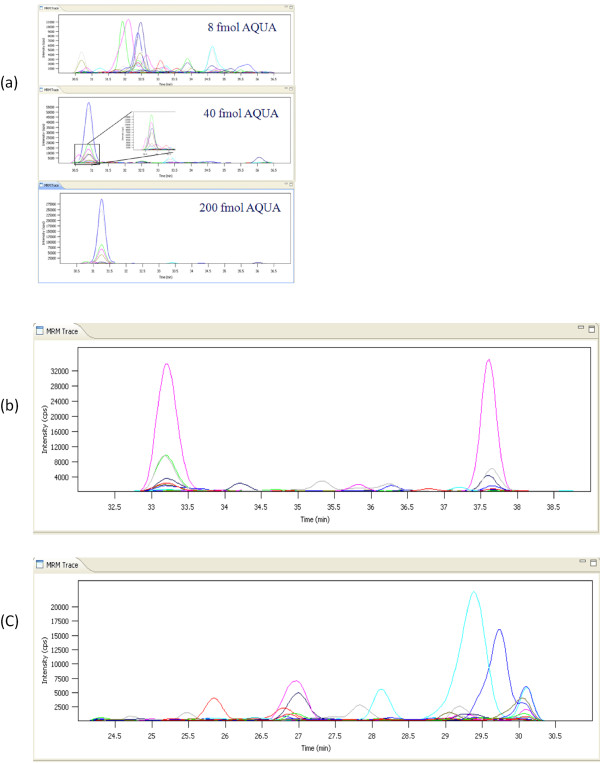
**Identified kinase peptide transitions in a cancer cell line**. (a) shows the smoothed heavy and light transitions of the kinase peptide AQLSTILEEEK in three different heavy peptide-spiked samples. (b) shows the kinase peptide IISIFSGTEK transitions. (a) and (b) show how ATAQS calculates the properties described in the 'identification of confirmed transitions' guided peptide detection in the sample, in spite of the strong interference. (c) shows an example of a decoy peptide RNVSTESIEF, which is above the FDR score cutoff of <1.2% in validation.

The data analysis took 25 minutes (including CPU, low activity system and IO) with a 2.33 GHz and 4 GB RAM hardware platform. Figure 7 shows the graphical summary reports for the kinase dataset. Compared to the synthetic light and heavy controlled yeast experiment, using a more complex and 'real' sample such as a cell line and a particularly challenging target protein group resulted in a less complete separation of decoy and target transitions. Figure 8(a) and 8(b) show the ATAQS-identified transitions guided by heavy pair transitions, even when there was strong interference in the transitions. Figure 8(c) illustrates a case where a decoy transition made it through with the FDR cutoff of 1.2%. With an FDR cutoff of 1.2%, 215 out of 450 peptides (50 peptides in nine cell line samples) were classified as correctly identified. Overall, the 215 identified peptides led to the detection of 41 out of 50 kinases in the tryptic digest of a human cell line subjected only to single dimensional chromatographic separation. The full list of 450 peptides in each sample, along with the score and associated error rate data, are provided at http://tools.proteomecenter.org/ATAQS/ATAQS.html.

## Conclusions

As a complement to the well-established discovery proteomic methods, targeted mass spectrometry based on SRM is becoming an important tool for the generation of reproducible, sensitive and quantitatively accurate data from biological samples. The method depends on the generation of target protein sets based on prior information and the one-time generation of verified mass spectrometric assays for each of the targeted proteins. The development of these assays depends on the optimal selection of peptides that represent the proteins on the target list and the optimal set of transitions for their detection in biological samples. Once developed, these assays can be continually applied across a multitude of studies.

The ATAQS pipeline and software provides a high throughput tool for organizing, generating and verifying transition lists and for the post acquisition analysis and dissemination of the data generated from applying the transition lists to studies of biological samples. ATAQS uses information from publicly accessible databases for the optimization of the protein and peptide target lists and for the optimization of a transition set. ATAQS is an open source software that enables hypothesis-driven research by providing the capability to generate candidate protein lists and measure candidate proteins across a large number of biological samples, and allows algorithm-developing scientists to further develop the steps in the ATAQS pipeline. For example, current ATAQS contains two different methods to generate decoys and to measure transition properties. GUI allows users to select one of these algorithms. Thus, computational biologists can extend the provided abstract class to add their own algorithms for transition generation steps and validation steps (Additional File 1: Plug-in your own algorithms). As needs arise, we plan to continuously expand on ATAQS functionalities (e.g., validation of quantification, support of SILAC type experiments, etc.). ATAQS is also available from Sourceforge.net (keyword search:Corra-ATAQS).

We expect that ATAQS will find wide application as targeted proteomics increases in use to support hypothesis-driven research across all fields of life science.

## Availability and Requirements

• Project name: ATAQS (Automated and Targeted Analysis with Quantitative SRM)

• Project home page: http://tools.proteomecenter.org/ATAQS/ATAQS.html and Sourceforge.net under Corra-ATAQS project.

• Operating System(s): Linux 2.6.18

• Programming language: Java, R. mysql

• Other requirements: Tomcat v 6.0.26 or higher, Java 1.6, Ant v1.7.1, mysql v 5.0.77, Firefox 3.6.x, Firegoose-0.8.259.xpi, Adobe Flash Player 10, R 2.11.1

• License: Apache 2.0

## Abbreviations

ATAQS: Automated and Targeted Analysis with Quantitative SRM; DFT: Discrete Fourier Transformation; FDR: False Discovery Rate; GEO: Gene Expression Omnibus; GO: Gene Ontology; GUI: Graphic user interface; HPRD: Human Protein Reference Database; LC-MS: Liquid Chromatography - Mass Spectrum; LDA: Linear Discriminant Analysis; LIMS: Laboratory Information Management System; PABST: Peptide Atlas Best SRM Transition; PATR: Peptide Atlas Transition Resource; PSI: Proteomics Standard Initiative; QQQ: Triple Quadrupole; ROC: Receiver Operating Characteristic; SRM: Selective Reaction Monitoring; TraML: Transition Markup Language;

## Authors' contributions

MB designed software architecture, oversaw the software development and test, and wrote this manuscript. SK and MC wrote ATAQS client and servlet codes and SK wrote decoy and validate transition modules. HR wrote firefox update and PIPE2 codes. DC wrote PABST and parsing TraML parser and validation module. LR contributed LDA analysis codes. PP measured SRM data. JC contributed disease specific curated dataset. ED and MB involved creating initial TraML format and ED presented to PSI and involved in coordinating committee review process. RA and RM oversaw and conceptualized the research project and contributed to writing the manuscript. All authors read and approved the final manuscript.

## Supplementary Material

Additional file 1 **It is ATAQS's step by step user guide using example dataset provided for this manuscript**. Click here for file
