# Supplementary material for: ATAQS: A computational software tool for high throughput transition optimization and validation for selected reaction monitoring mass spectrometry
Source: BMC Bioinformatics. 2011 Mar 18;12:78. doi: 10.1186/1471-2105-12-78 (PMC3213215; doi:10.1186/1471-2105-12-78)
Supplement: Additional file 1 — It is ATAQS's step by step user guide using example dataset provided for this manuscript. [file 1471-2105-12-78-S1.PDF]

ATAQS v1.0 User's Guide

Revolutionizing science. Enhancing life.

ATAQS: A computational software tool for high throughput transition optimization and validation for selected reaction monitoring mass spectrometry

*Mi-Youn K. Brusniak[1], Sung-Tat Kwok[1], Mark Christiansen[1], David Campbell[1], Lukas Reiter[2], Paola Picotti[2], Ulrike Kusebauch[1], Hector Ramos[1], Eric W. Deutsch[1], Jingchun Chen[3], *§Robert L. Moritz[1], *§Ruedi Aebersold[2,4,5]

[1]Institute for Systems Biology, Seattle, WA, USA; [2]Institute of Molecular Systems Biology, ETH Zurich, Zurich, Switzerland; [3]Information Warehouse, the Ohio State University Medical Center, OH, USA; [4]Competence Center for Systems Physiology and Metabolic Disease, ETH Zurich, Zurich, Switzerland; [5]Faculty of Science, University of Zurich, Zurich, Switzerland;

*Corresponding authors, § this work was performed equally between the two groups.

Abstract

Background

Since its inception, proteomics has essentially operated in a discovery mode with the goal of identifying and quantifying the maximal number of proteins in a sample. Increasingly, proteomic measurements are also supporting hypothesis-driven studies, in which a predetermined set of proteins is consistently detected and quantified in multiple samples. Selected reaction monitoring (SRM) is a targeted mass spectrometric technique that supports the detection and quantification of specific proteins in complex samples at high sensitivity and reproducibility. Here, we describe ATAQS, an integrated software platform that supports all stages of targeted, SRM-based proteomics experiments including target selection, transition optimization and post acquisition data analysis. This software will significantly facilitate the use of targeted proteomic techniques and contribute to the generation of highly sensitive, reproducible and complete datasets that are particularly critical for the discovery and validation of targets in hypothesis-driven studies in systems biology.

Result

We introduce a new open source software pipeline, ATAQS (Automated and Targeted Analysis with Quantitative SRM), which consists of a number of modules that collectively support the SRM assay development workflow for targeted proteomic experiments (project management and generation of protein, peptide and transitions and the validation of peptide detection by SRM). ATAQS provides a flexible pipeline for end-users by allowing the workflow to start or end at any point of the pipeline, and for computational biologists, by enabling the easy extension of java algorithm classes for their own algorithm plug-in or connection via an external web site.

This integrated system supports all steps in a SRM-based experiment and provides a user-friendly GUI that can be run by any operating system that allows the installation of the Mozilla Firefox web browser.

Conclusions

Targeted proteomics via SRM is a powerful new technique that enables the reproducible and accurate identification and quantification of sets of proteins of interest. ATAQS is the first open-source software that supports all steps of the targeted proteomics workflow. ATAQS also provides software API (Application Program Interface) documentation that enables the addition of new algorithms to each of the workflow steps. The software, installation guide and sample dataset can be found in http://tools.proteomecenter.org/ATAQS/ATAQS.html

ATAQS is an open source software Licensed under the Apache License, Version 2.0 and it's source code, demo data and this guide can be downloaded at the http://tools.proteomecenter.org/ATAQS/ATAQS.html.

This documentation was prepared by Mi-Youn Brusniak (mbrusniak@systemsbiology.org).

1. Introduction

As a complement to the well-established discovery proteomic methods, targeted mass spectrometry based on SRM is becoming an important tool for the generation of reproducible, sensitive and quantitatively accurate data from biological samples. The method depends on the generation of target protein sets based on prior information and the one-time generation of validated mass spectrometric assays for each of the targeted proteins. The development of these assays depends on the optimal selection of peptides that represent the proteins on the target list and the optimal set of transitions for their detection in biological samples. Once developed, these assays can be continually applied across a multitude of studies.

The ATAQS pipeline and software provides a high throughput tool for organizing, generating and verifying transition lists and for the post acquisition analysis and dissemination of the data generated from applying the transition lists to studies of biological samples. ATAQS uses information from publicly accessible databases for the optimization of the protein and peptide target lists and for the optimization of a transition set. ATAQS is open source software that enables data-driven researchers to generate candidate protein lists and measure candidate proteins across a large number of biological samples, and allows algorithm-developing scientists to further develop the steps in the ATAQS pipeline. As needs arise, we plan to continuously expand on ATAQS functionalities (e.g., validation of quantification, support of SILAC type experiments, etc.).

We expect that ATAQS will find wide application as targeted proteomics increases in use to support hypothesis-driven research across all fields of life science. ATAQS is a single, user-friendly, informatics framework, that is simple to use and fully customizable, for the enabling of SRM-based proteomic workflows of any size, able to guide the user seamlessly from MS data generation, through data processing, visualization, and statistical analysis steps, to verify proteins of interest in biological samples. This is a user guide for ATAQS v1.0 software.

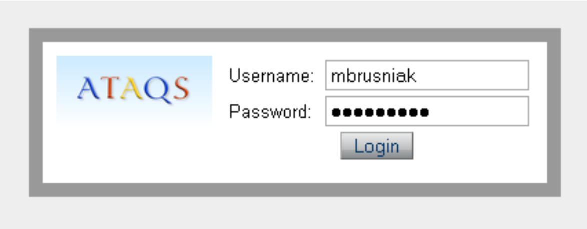

2. Login

Website: Ask administer in your institution which server the ATAQS is deployed to and ask ATAQS admin to add your account. For this guide, we will

use ATAQS account.

The URL should be something like the following. http://moog.systemsbiology.net:8080/ATAQS. If you are using ATAQSDemo.systemsbiology.net for "read only" demo, you can type ATAQS in Username and Demo2010 in password and admin in Username and admin@isb2010 in password for Admin page demo in the following section.

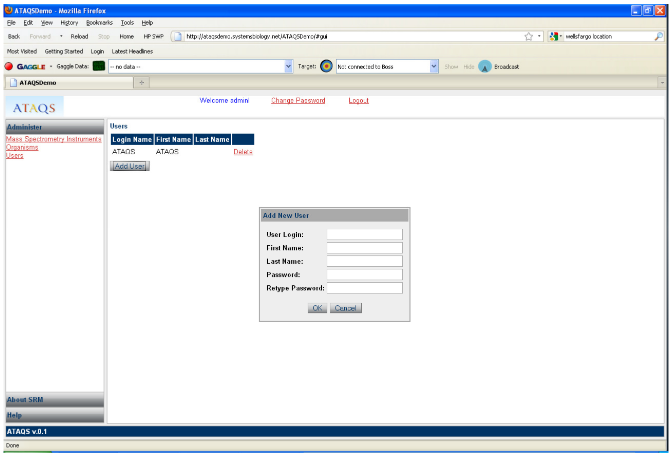

3. Admin page

If you are logged in as admin, ATAQS leads to admin page, where you can configure for all users.

You can select **Users** in **Administers** panel, the main panel will display the current 'Users' list. When you click **"Add Users"** button, the **Add New Users** panel will be opened as shown in the left figure.

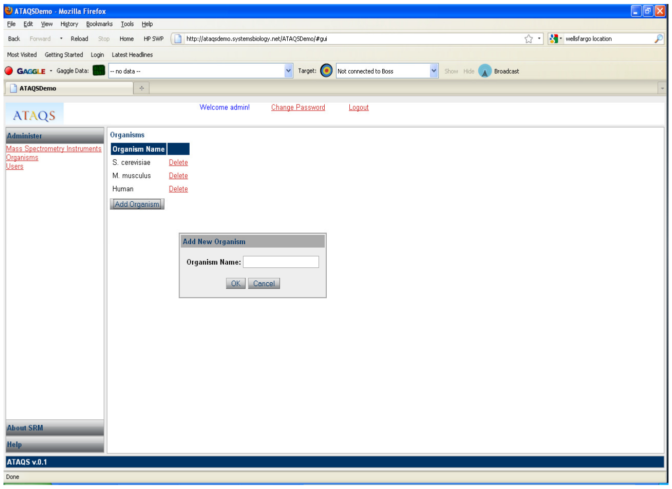

Administrator also can add available organisms. ATAQS has a step to connect publically available website PIPE2 (Protein Information and Property Explore) using the administrator defined organism. Thus, we advise administrator to see how the organism was named by PIPE2. Administrator can add organism by selecting **Organisms** in **Administrators** panel shown in the left figure.

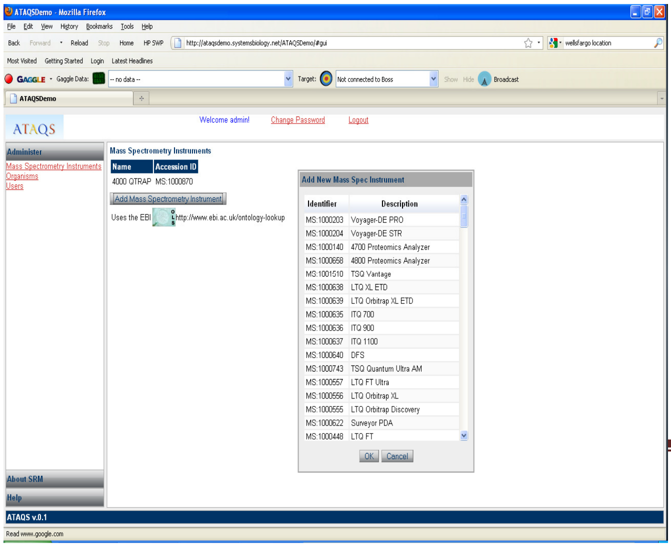

Administrator also can add available mass spectrometry Instruments by selecting **Mass Spectrometry Instruments** option in **Administrators** panel. When you click "Add Mass Spectrometry Instrument" button, ATAQS connects to EBI website for http://www.ebi.ac.uk/ontology-lookup service to get Identifiers for all available mass spectrometry. Using the controlled vocabulary is necessary to generate resulted

file to be exchangeable to community when user decided to publish their validated transitions at the end of ATAQS pipeline.

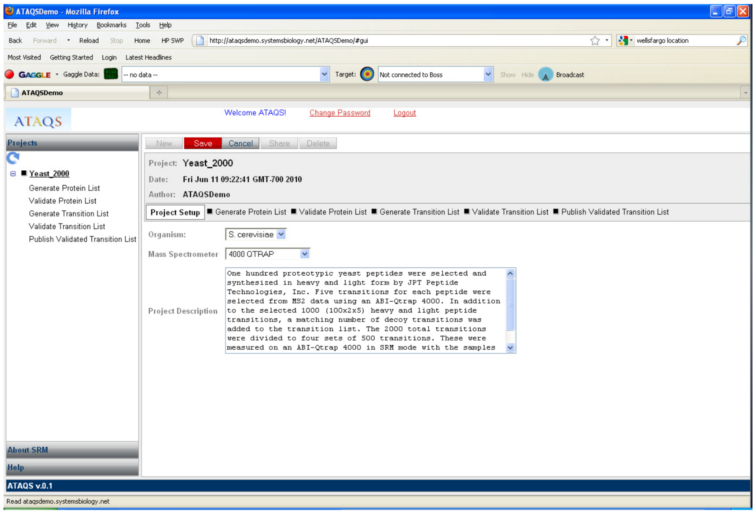

4. Project Setup Step

Creating and sharing project. After user login using their user account, ATAQS pipeline will show as shown in the left panel. Click “**New**” button in the top panel will ask the project name. You can type in Yeast_2000. Then it will go to “**Project Setup**” panel. User can select the organism and mass spectrometry for the project by using pull down options. There is “**Project Description**” text box that user can type overview of the experiment. You

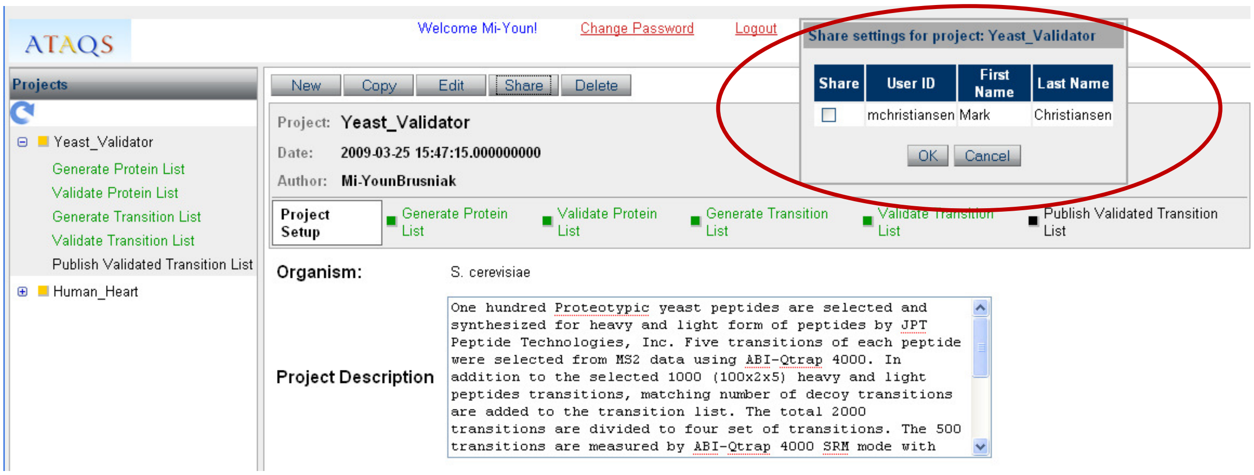

can select organism and mass instrument as shown in the figure and click “**Save**” button. ATAQS software is designed to serve institution where several collaborators are working with similar SRM experiments. Thus, ATAQS provide a way to share the projects with collaborators. When user select “**Share**” button, “**Share setting for Project:** *your named pro*ject” panel (circled in red) and list of users. You can select collaborators that you would like share the project. The current implementation of sharing project provides collaborators to access your project read-only mode. More specifically, collaborator cannot modify your steps. Create project by click “**New**” button and type *Yeast_2000*. Select Yeast in the Organism and select 4000 QTRAP for Mass spectrometry and you can write some description of the project. This project is an example of ATAQS paper using 100 heavy and light synthesized yeast peptides with 3 dilution series of three

different background samples (glyco captured human plasma, C. elegans and Leptospira interrogans extracts). Then click “**Save**” button.

5. ATAQS Pipeline status indicator

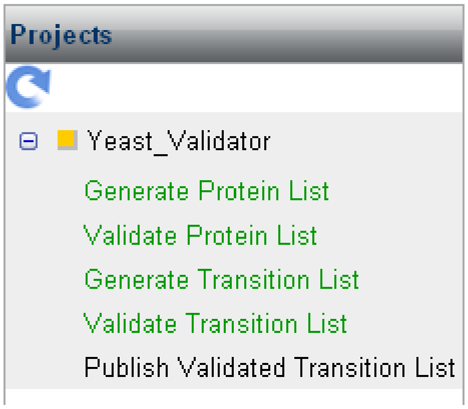

ATAQS provides a quick way to help users to glace each project and steps status. In the left “**Projects**” Panel, it list all the projects that are you created and collaborators are sharing. The square button beside of each project indicate whether the projects are completed (“green”) or not started (“black”) or in the middle of the process (“yellow”). For example, left figure has yellow square box beside the project Yeast_Validator and there are total of five steps and the first four steps are completed but not the last step was done. Thus, the project status is yellow as not totally completed project. ATAQS lists the step in the right side panel where user can click to go to the step. In the panel, it also shows the status of the step. The step status is synchronized with the step status in “**Projects**” panel. The color of square buttons and step color indicates the each step status as shown in the left panel.

6. Generate Protein List

Select “**Generate Protein List**” step in the top panel. You will see “Step1: Generate Project List” step.

There are two ways to populate protein list in this step. If there are institution wide database, administrator can add the protein list in tsv format to ATAQS site so all the users are accessible to the same database. As example, this version of ATAQS contains three bioinformatically curated disease-specific protein candidate lists: (1) Prostate tumor containing 1055 proteins, (2) Type II diabetes containing 954 proteins and (3) Breast cancer-related human kinase signaling containing 32 proteins. You can select one of the three database or administrator installed institution specific database from the pull down menu of “**Existing Protein List**” option. Or you can load your own protein list by clicking “**Edit**” button and click “**Browser**” then select *ATAQS_YeastProteinList.csv* and click “**upload”** button. The summary of protein uploaded will be shown like the picture. In this step, if you had already some of protein, ATAQS will merge them with unique protein entry list.

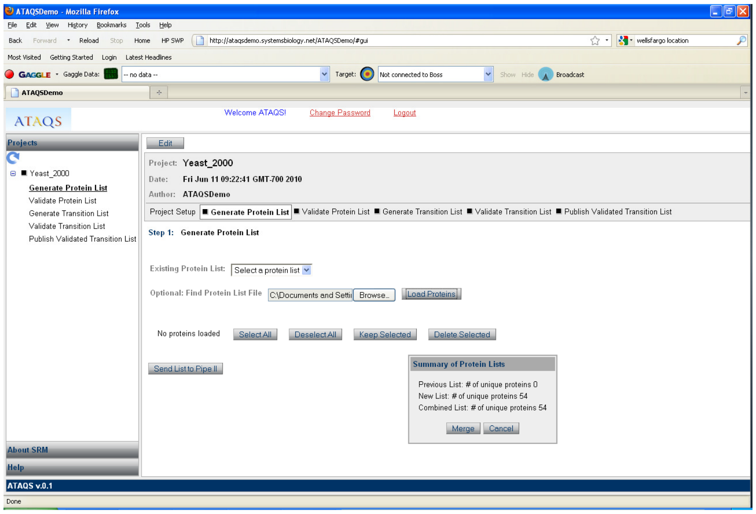

7. Setting up connection to other website

Make sure you are using firefox (3.x versions) browser and installed Firegoose using Firegoose-0.8.208.xpi or higher from http://gaggle.systemsbiology.net/docs/geese/firegoose/install/ as indicated in ATAQS installation guide. Go to Firefox and select pull down menu of firegoose. Then select "Add Custom Website Handler". It will bring the panel shown in the left side. You will fill out the name and URL. This is the way you can receive back any data from public website. The current version of ATAQS uses three publically available website, PIPE2, MRMAtlas and TraML uploading website for MRMAtlas backend repository.

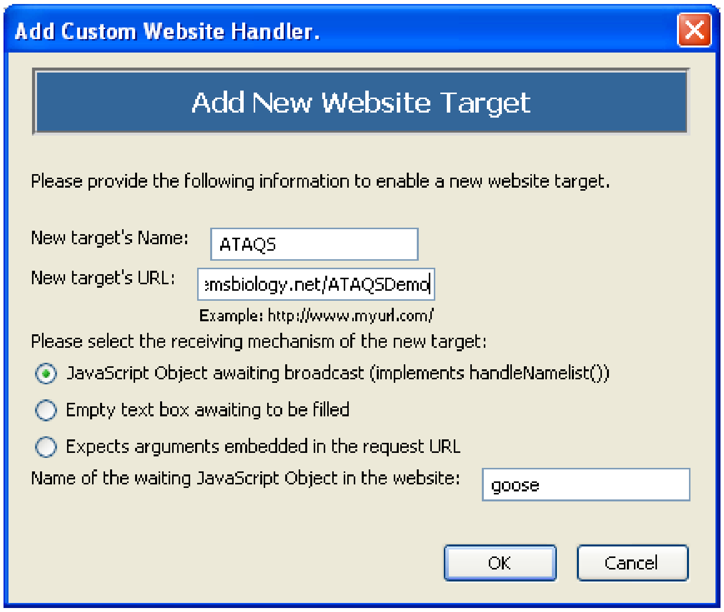

8. Investigating Protein Properties using PIPE2

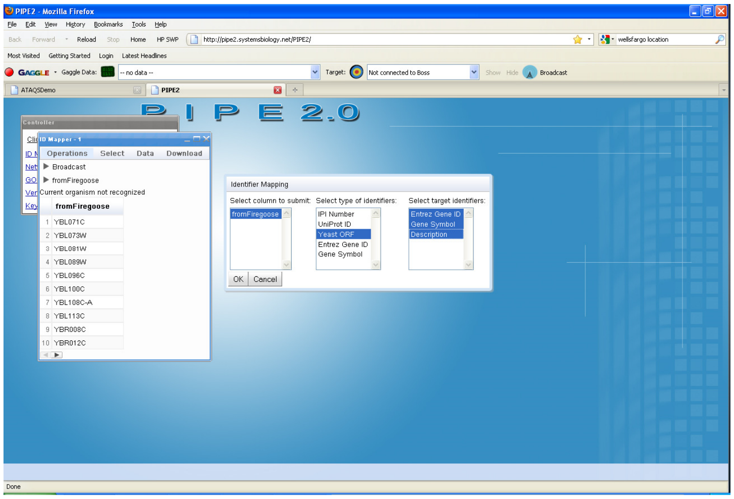

Go back to ATAQS "**Generate Protein List**" step. You can investigate the uploaded protein list properties further using PIPE2. After investigating Protein list, you can expend or remove protein list.

Click "**Select All**" Button then click "**Send List to PIPE II**" button in the bottom of the panel. The list will be sent to PIPE2 website by firegoose. New tab in the firefox browser will be open and your protein list will be displaced as indicated in the right side figure. You can use PIPE2 tools (please refer PIPE2 tutorial guide for detailed PIPE2 functionality) to generate Protein-Protein interaction maps as shown in the figure. You can select subset of proteins from the network view and send back to ATAQS protein list as next page figures. For this exercise, we will broadcast back the same list.

In the PIPE2, IDMapper PIPElet, Select *fromFiregoose* in **Data** pull down menu and select “Firegoose” in

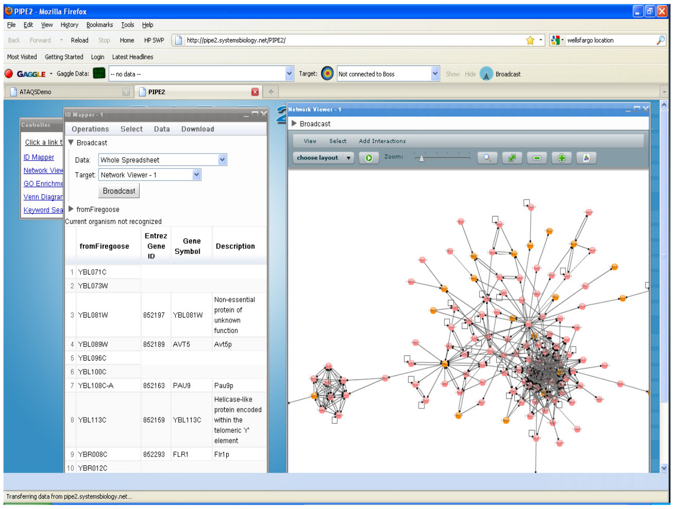

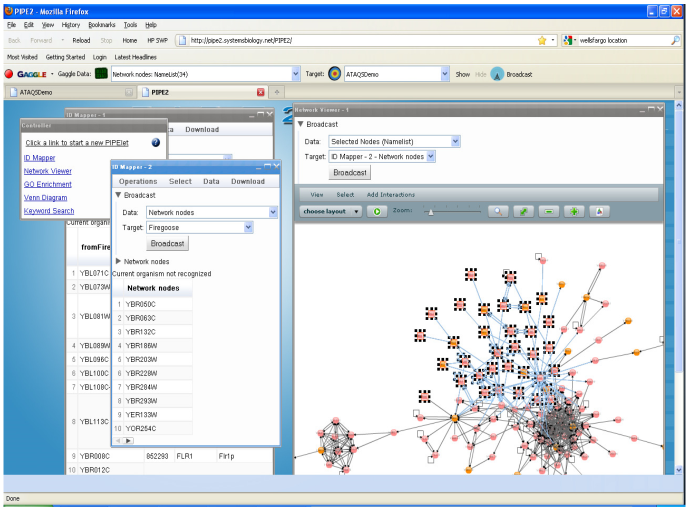

Target pull down menu and click “**Broadcast**”. Select “ATAQS” in the Firegoose menu in your Firefox

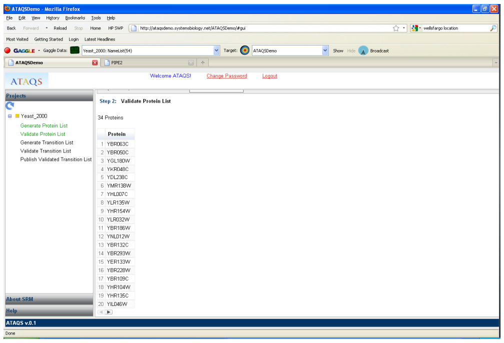

browser and click “**Broadcas**t” button of the Firegoose. Your new protein list (in this example, the same protein list) will be back to ATAQS “**Validate Protein List**” panel shown below. Note that “**Generated Protein List**” step is in green and “**Validate Protein List**” step is in yellow.

When you click “**Save**” button. The “**Validate Protein List**” step turns to green to indicate that this step is also completed for this project.

9. Generating Transition List.

ATAQS “**Generate Transition List**” allows either uploading your optimized transition list or obtaining best observable peptides from MRMAtlas while considering user weighted penalty factor. In this manual, you will demonstrate both ways. First, click “**Edit**” button and click “**MRMPeptideAltas**”

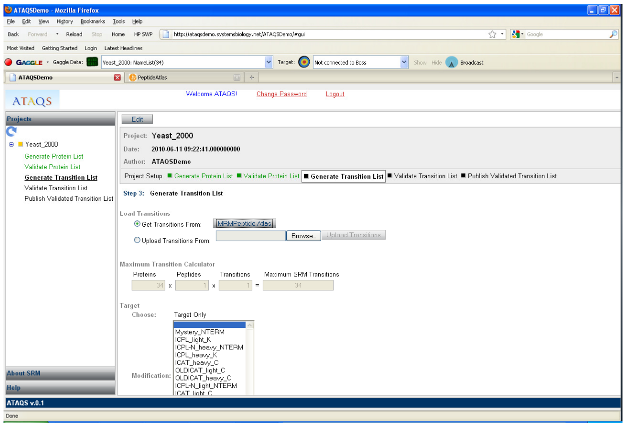

button as shown in the left figure. It will bring MRMPeptideAtlas page as shown in the next figure. Notice that your proteins are already filled in that page. Select *YeastPublic2010-02* in PABST (PeptideAtlas Best Transition) build and select 3 for number of peptide per proteins. Exam all the options in getting number of transitions and also options to excluding peptides with certain amino acid.

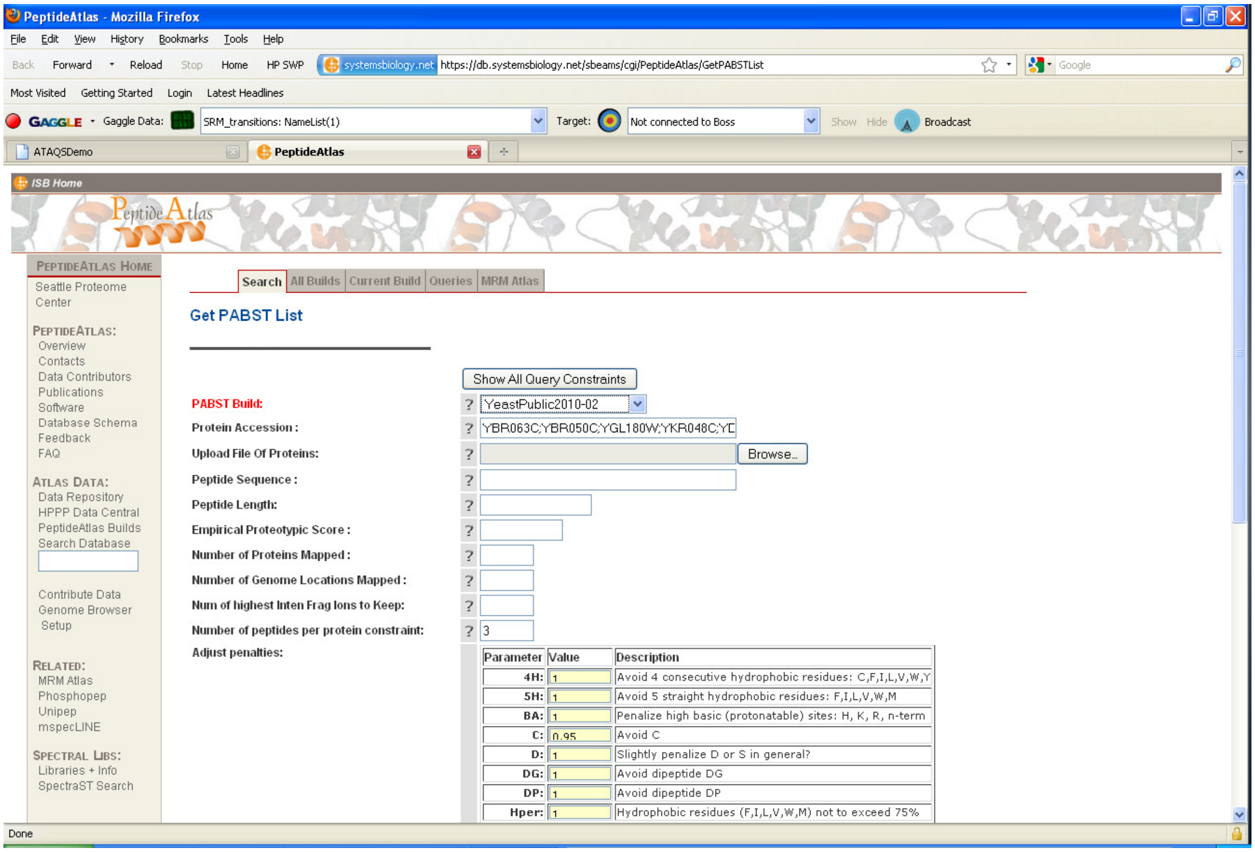

When you click "Get Transition", it will show transitions. You can again broadcast back the transitions by clicking "Broadcast" button in Firegoose, those transition list will be back to your project in ATAQS as shown below figure.

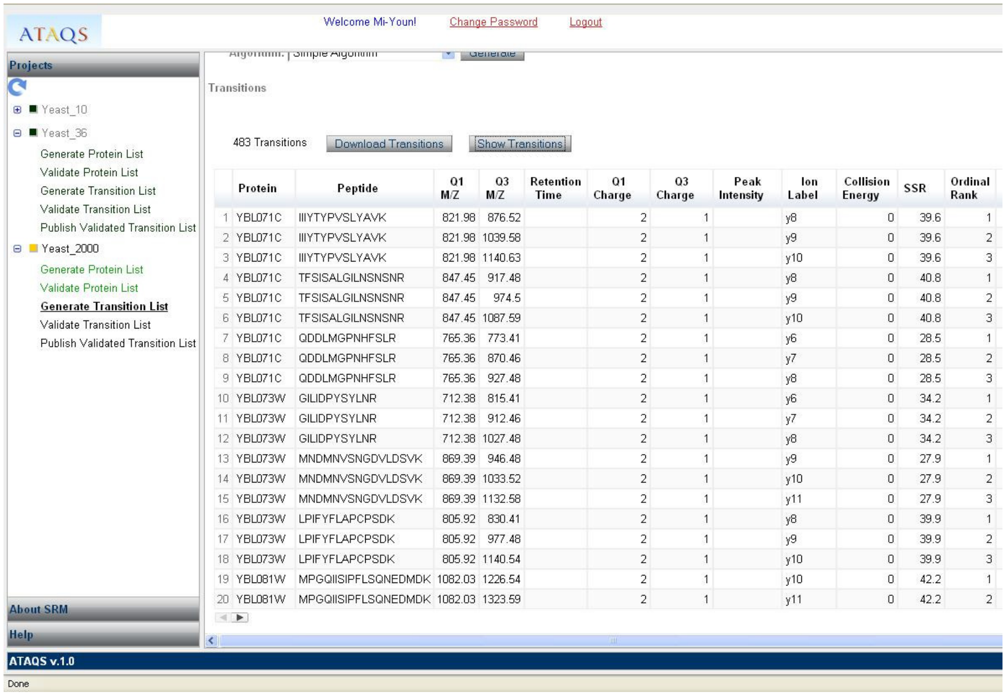

As mentioned earlier, ATAQS can also just take transition list from users by Clicking "**Browser**" and select ATAQS_D2_Transition_TargetOnly.csv file. Then click "**upload**", the uploaded transition will replace the transitions we got from MRMPeptide Atlas. ATAQS allows latest transitions either from user upload or from MRM PeptideAtla to be the current transitions. As described in the paper, you need decoy to validate your transition detection. Thus, this step allows generating additional transitions to append to your current transition. If transitions are generated from MRM Peptide, you may need heavy peptide (e.g., AQUA peptide) pair transitions to measure both heavy and light transition in your biological sample. Or you optimized transition using heavy peptides, and then you need light pair to measure in your sample as well. Thus ATAQS allows several options to append transitions to your current transitions. Entire selection of those options are shown in the figure.

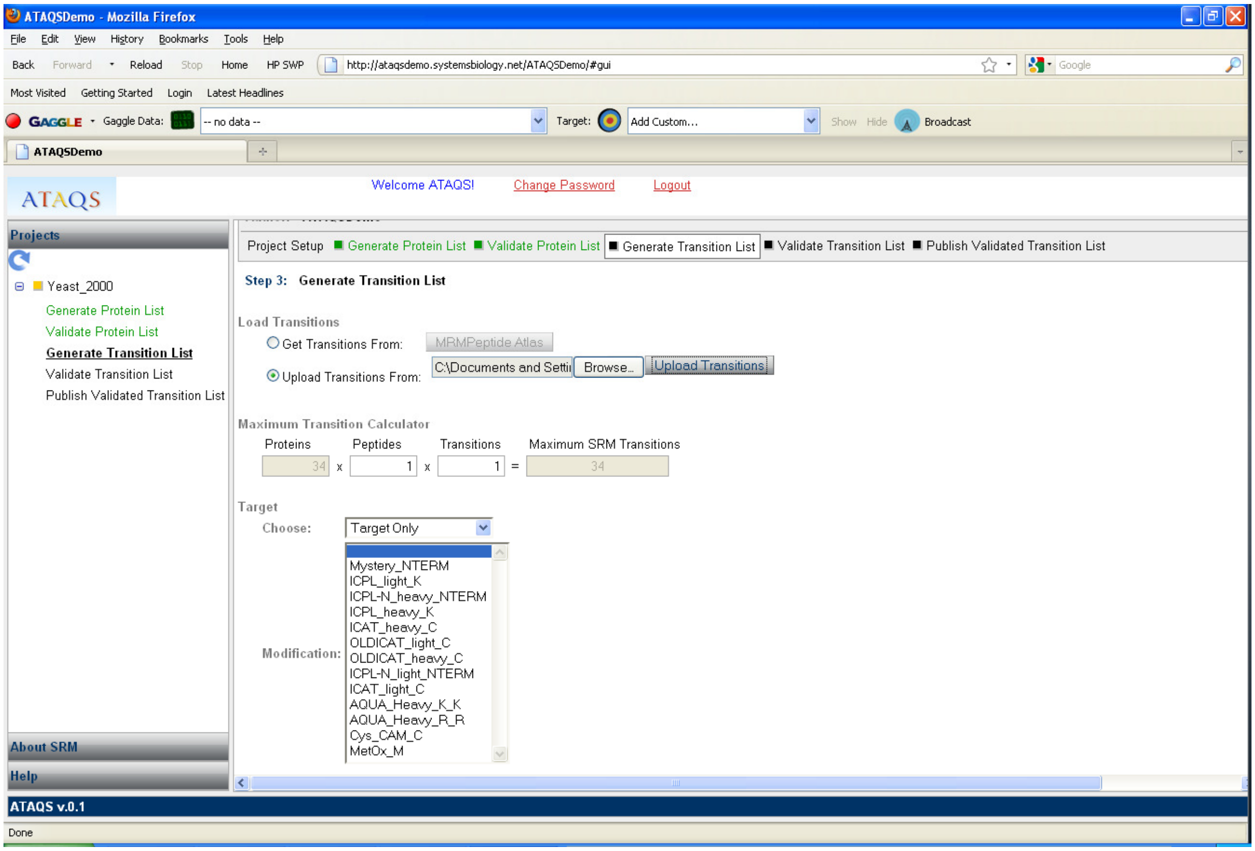

As described in the paper, ATAQS needs decoy transitions to score your measured transitions with your sample. You can easily extend ATAQS software to add additional decoy algorithms. Current version of ATAQS comes with two decoy generating algorithms described in the paper. In this example, the uploaded yeast transitions have heavy and light transitions. Thus, we will use "**Target Only**" option in Target section and select "Simple No Overlap Algorithm" for "Decoy Generation" option. Then click "**Generate**" button. Notice the step goes to "**yellow**" state and the algorithm generating process were initiated in one of your institution computing node. ATAQS designed to separate computing intensive processed to be outside of servlet so ATAQS web application would not be locked.

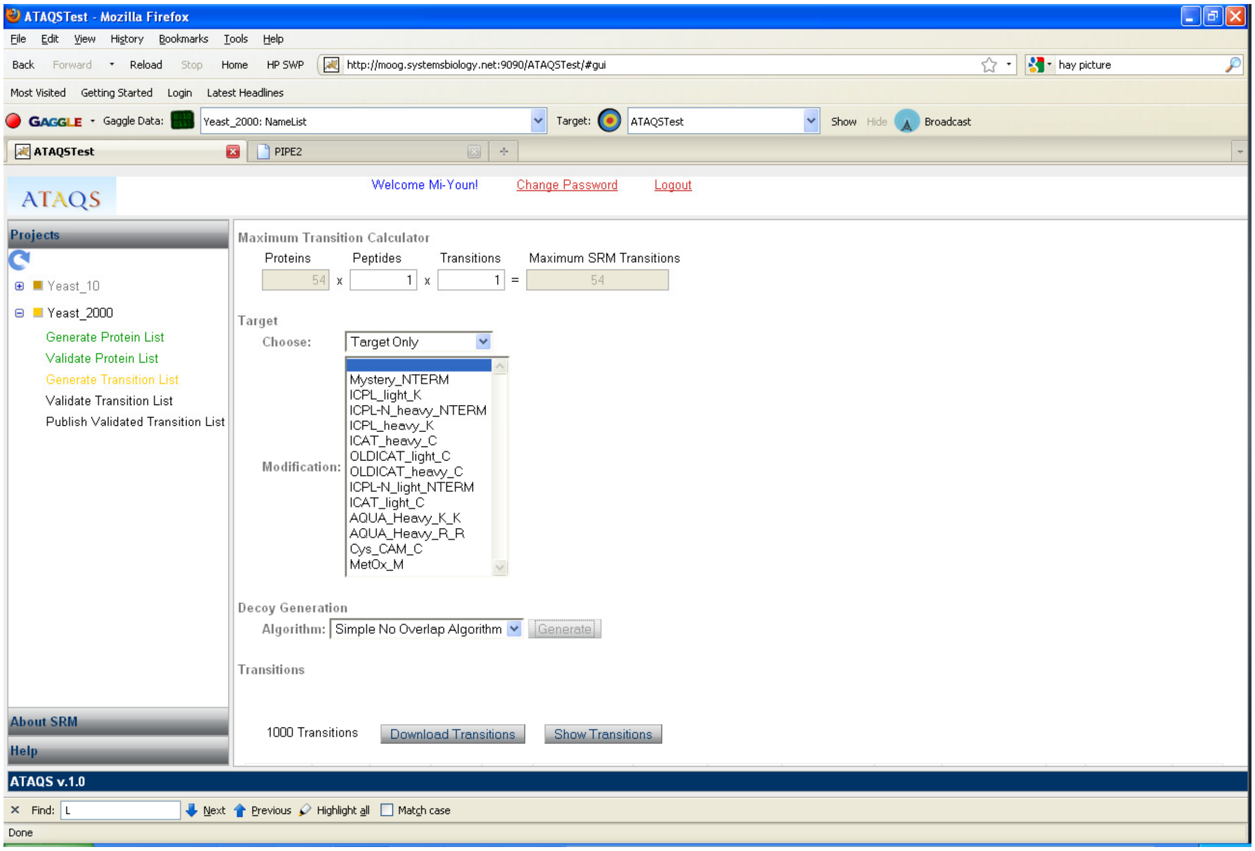

In this example, when the process is finished, total 2000 transitions were generated. Then the transitions can be downloaded by click "**Download Transition**" button to download the transition in your desktop. The transition list can be used in measuring in your sample.

10. Validating Transition List.

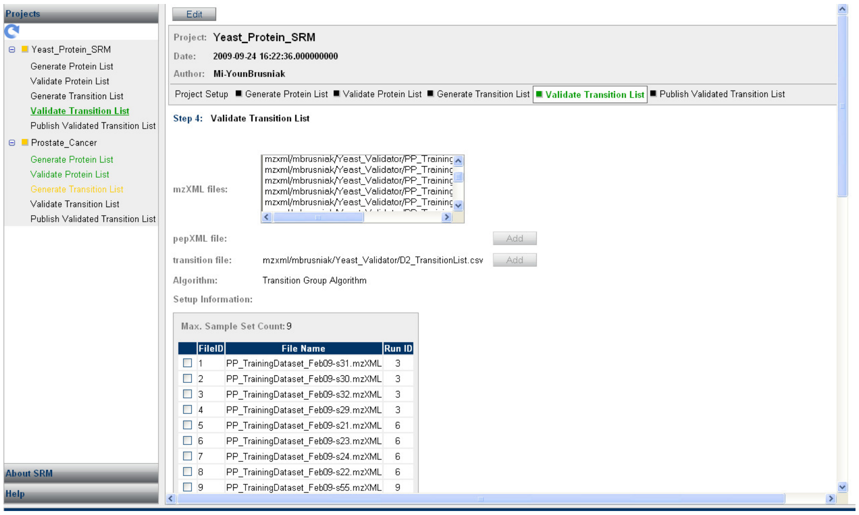

In this example, we split 2000 transitions to four 500 transition and prepared 9 samples as described in the paper (3 dilutions and 3 kind biological samples). Thus total 36 (4 transition set per sample) LC-MS were run in 4000 QTrap MRM mode. It's daunting for users to go through and validate each transition to see whether they detected the peptide or

not manually. Thus, the current ATAQS version has mProphet module to assign score to the peptide based on discriminate properties between decoy and target transitions. Since the transitions were split to four, ATAQS allows user to group those samples together. First, ATAQS can take either mzXML or mzML files. You select “**Add**” buttons to select all 36 mzXML files. Select files 5-8, 13-16, 21-24, 29-32, 37-40, 45-48, 53-56, 61-64, 69-72 numbered mzXML files. Click “**Max. Sample Set Count**” and type *9*. Then assign each four set of mzXML to each “Run ID” to indicate which samples belong to one. As mentioned in the paper, users can optimize their own transitions so ATAQS allows uploading transition list which matches their measured transitions. The transition list will be uploaded by clicking “**Add**” in transition file and select D2_TransitionList.csv. ATAQS allows computational biologists to extend any of algorithms to be part of ATAQS. In this version, we provide two algorithms. For this example, click “**Transition Group Algorithm**” in Algorithm section then click “**Run Validator**”. This step takes a few minutes to finish up. Similar to generating transitions, mProphet module will be running in one of your institution distributing node. When “Validate Transition List” step is completed, the step will turn to green and ATAQS displays the graphical summery of the dataset. There will be a drop down menu to show “**ROC**”, “**FDR**” and “**Separation Bar Chart**”. You can download the scored transition to see which transition group has higher validated score. For example, IAWEALAVER_1 in downloaded file Yeast_2000_top_pg.xls means the peptide is detected in Run ID sample group 1.

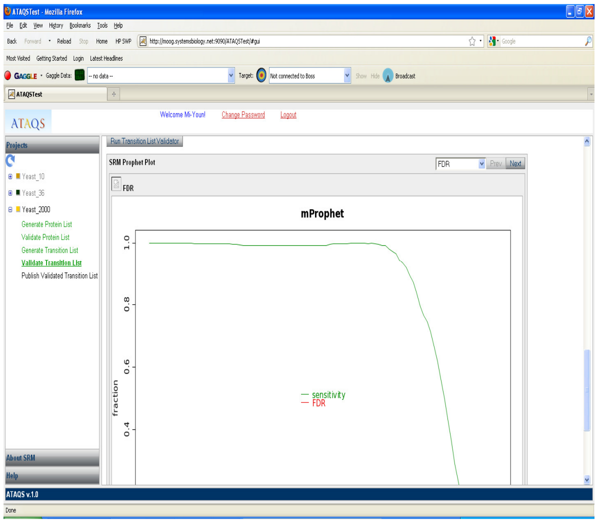

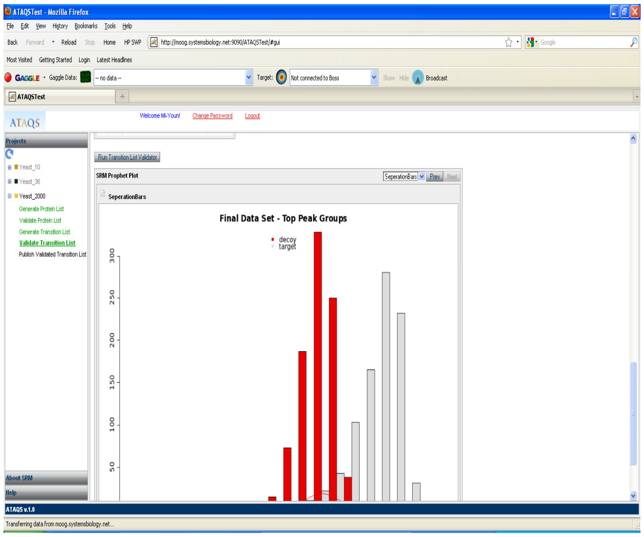

11. Publishing Validated Transitions.

As an optional step, all optimized and validated transitions can be made available to the community, so that the data can be used for SRM-driven biological research such as biomarker validation. Or simply store your final transition list in standard format in your institution. ATAQS also introduces a new proposed file format called TraML (Transition Markup Language) as a common data exchange format for validated transition information as described in ATAQS paper. ATAQS helps create TraML format files for exchange of validated transition information and if the user chooses to publish their data, ATAQS provides an easy way to upload user-created TraML files to public SRM databases, such as MRMAtlas.

In the last step of ATAQS "Publish Validate Transition List", you can fill out a few contact information for author of the generated TraML file. Simply click "Create TraML" will generate TraML file with your project name and author name. In this case, Yeast_2000_Mi-YounBrusniak.TraML will be generated and you can download and modify the file or simple save the file. You can browse the content of TraML using various xml viewers including Firefox browser shown below.

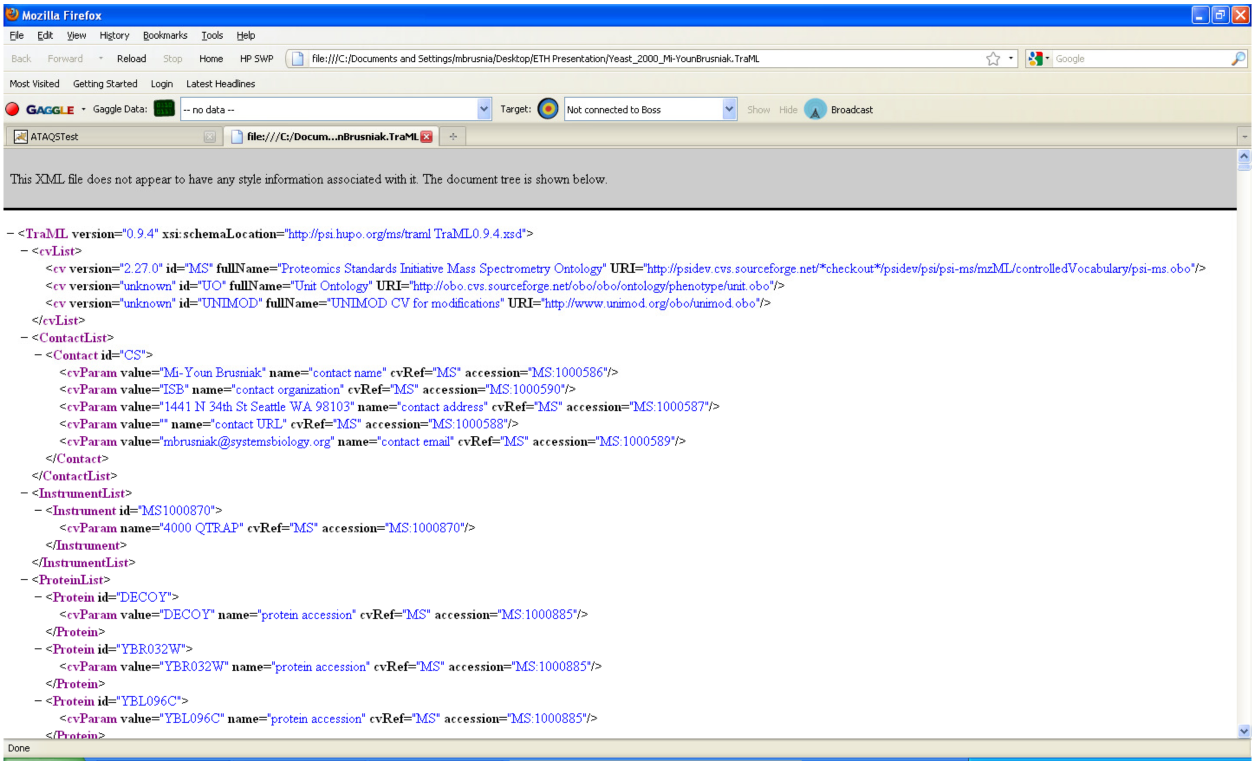

When you decide to publish your TraML file to MRMAltas website, you can upload the file and select "Publish" button. ATAQS will validate the TraML based on current TraML xml schema for well formed and then upload MRMAtlas designated website. When the files are successfully uploaded to MRMAltas, ATAQS will generate "**Event Notification**" Panel to indicate success as shown in the figure.

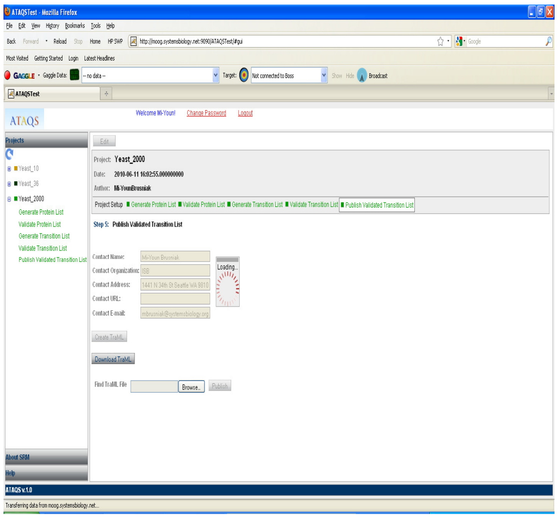

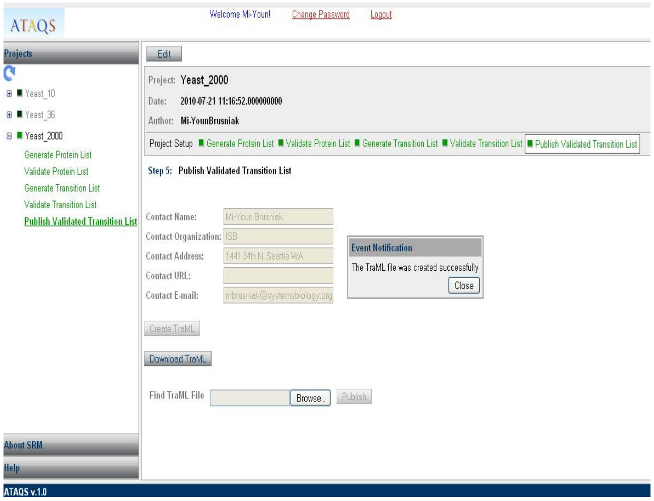

12.. Plug-in your own algorithms

Additional to use of ATAQS as a SRM pipeline as it is, computational biology can add their own algorithms to ATAQS since it is an open source software. For example, if a biologist has another way to generate decoy algorithms or measuring transition properties (e.g., current ATAQS Transition Group Algorithm measures intensity from Apex of transition rather than area), computational biologist can extend abstract class called org.systemsbiology.apps.validator.AbstractValidationAlgorithm class to implement or overwrite functions to determine background noise and intensity measurements. When the algorithms are implemented and compiled using ATAQS provide building command, the algorithm is ready to be used. Next, administrator needs to add the algorithm name to "Algorithm table" in the ATAQS database to be part of pull down menu selection in Validator step.

13. Conclusion

As a complement to the well-established discovery proteomic methods, targeted mass spectrometry based on SRM is becoming an important tool for the generation of reproducible, sensitive and quantitatively accurate data from biological samples. The method depends on the generation of target protein sets based on prior information and the one-time generation of validated mass spectrometric assays for each of the targeted proteins. The development of these assays depends on the optimal selection of peptides that represent the proteins on the target list and the optimal set of transitions for their detection in biological samples. Once developed, these assays can be continually applied across a multitude of studies.

The ATAQS pipeline and software provides a high throughput tool for organizing, generating and verifying transition lists and for the post acquisition analysis and dissemination of the data generated from applying the transition lists to studies of biological samples. ATAQS uses information from publicly accessible databases for the optimization of the protein and peptide target lists and for the optimization of a transition set. ATAQS is open source software that enables data-driven researchers to generate candidate protein lists and measure candidate proteins across a large number of biological samples, and allows algorithm-developing scientists to further develop the steps in the ATAQS pipeline. As needs arise, we plan to continuously expand on ATAQS functionalities (e.g., validation of quantification, support of SILAC type experiments, etc.).

We expect that ATAQS will find wide application as targeted proteomics increases in use to support hypothesis-driven research across all fields of life science.
